# Mathematical modeling of the immune system recognition to mammary carcinoma antigen

**DOI:** 10.1186/1471-2105-13-S17-S21

**Published:** 2012-12-07

**Authors:** Carlo Bianca, Ferdinando Chiacchio, Francesco Pappalardo, Marzio Pennisi

**Affiliations:** 1 Dipartimento di Scienze Matematiche, Politecnico di Torino, Torino, Italy; 2 Dipartimento di Ingegneria Informatica e delle Telecomunicazioni, Università di Catania, Catania, Italy; 3 Dipartimento di Scienze del Farmaco, Università di Catania, Catania, Italy; 4 Dipartimento di Matematica e Informatica, Università di Catania, Catania, Italy

## Abstract

The definition of artificial immunity, realized through vaccinations, is nowadays a practice widely developed in order to eliminate cancer disease. The present paper deals with an improved version of a mathematical model recently analyzed and related to the competition between immune system cells and mammary carcinoma cells under the action of a vaccine (Triplex). The model describes in detail both the humoral and cellular response of the immune system to the tumor associate antigen and the recognition process between B cells, T cells and antigen presenting cells. The control of the tumor cells growth occurs through the definition of different vaccine protocols. The performed numerical simulations of the model are in agreement with in vivo experiments on transgenic mice.

## Background

The immune system (IS) is a complex system of organs, cells and molecules whose main task is to protect living organisms from external pathogens such as viruses and bacteria. Nevertheless the effectiveness of the IS in tumors disease is nowadays under discussion among biologists and physicians. As stated by the immunosurveillance theory [1,2], biotechnology engineered naked mice (mice without immune system) show the developing of multiple variants of malignant tumors that are not usually visible in wild mice, thus suggesting that the immune system plays an important role also against tumors. Indeed most mutated malignant cells are recognized and eliminated by immune system mechanisms right after their birth, and tumors that usually arise are indeed poorly immunogenic tumors, originating from malignant cells which escape from immune surveillance. Some tumors are caused by exogenous factors (e.g., smoke for lung cancer), and the elimination of the exogenous cause would in theory prevent the risk of developing the tumor. However many other tumors are caused by endogenous factors and their developing cannot be easily predicted and controlled. Among human cancers, the mammary carcinoma represents a major cause of concerns in women, since it belongs to the class of endogenous cancers which escape immunosurveillance of the IS.

The risk of appearance of mammary carcinoma is usually estimated by analyzing the family history of cancer, and breast cancer screening in young women is highly recommended since the achievement of earlier diagnosis could greatly improve the outcomes of the treatment. Strong family history of cancer usually entitles higher risks of developing the tumor, thus suggesting that tumor hereditary is encoded into the DNA. Some gene tests such as the genetic screening for the BRCA genes [3] are nowadays possible and may determine the risk of cancer. Indeed the analysis of the genome of individuals will be useful to better estimate the risk of cancer.

Biologists and physicians are exploring novel immunopreventive treatments that can avoid the development of breast cancer in patients with high risks of malignant cell mutations. Among others, Lollini et al. [4] have developed a cellular vaccine, called Triplex, which is able to elicit complete prevention of mammary carcinogenesis in HER-2/neu transgenic mice. Triplex combines three different elements (the tumor antigen with two adjuvants) that stimulate the immune system response with different actions [4]. Vaccine cells have been engineered to present and release the tumor associated antigen p-185 (that is also the oncogene of the tumor) with the addition of Allogeneic MHC-class I molecules to easier recognizing by cytotoxic T cells. Moreover, thanks to transduction of interleukin-12 genes, they release interleukin-12 molecules that have a broad range of costimulatory functions in boosting the immune response against tumors.

It is worth stressing that differently from the vaccine for virus or bacteria, cancer vaccines require repeated administration for the the entire life of the host. This is due to the low antigenicity of the cancer cells, the capability to escape the immune system surveillance. Moreover present cancer immunoprevention research is unable to find better vaccines able to assure complete, long-term protection.

The repeated administration of the vaccine, realized with the aim to increase the antigenicity of the tumor associated antigens, maintains the immune system response *ready against newborn cancer cells*. However, even if vaccines are usually less toxic than standard drugs, uncontrolled administration of the vaccine can induce undesirable effects such as autoimmune diseases. Therefore the optimization of the vaccination protocol constitutes a fundamental and open problem.

In the in vivo experiments it is not usually possible to reach an optimum vaccination protocol that maximizes the efficacy of the tumor depletion on the one hand and minimizes the risk of side effects on the other hand, because of the large variability cases. Indeed vaccination protocols are usually determined heuristically basing on best practice and previous experience. Moreover the cost of in vivo experiments can be prohibitive.

In order to understand whether it was possible to gain complete prevention of mammary carcinogenesis with fewer injections, a (multi) agent-based model named SimTriplex [5] has been developed. It is worth noticing that SimTriplex has been also employed for other pathologies [6-10]. However agent-based models do not allow the development of asymptotic analysis of the competition and an easy investigation of parameters' space.

Different mathematical tools have been developed in order to model complex biological systems and among others, immune system-cancer competition. The most famous approach is the ODE-based model where the overall system is decomposed in different cell populations whose time evolution is depicted by solutions of a nonlinear ODE system (nonlinear terms take care of the interactions among two or more cell populations), see paper [11] for a review of ODE models available in the literature and [12] for a comparison between ODE models with and without delay.

Kinetic theory models have been also proposed for the immune system-cancer competition. These models consist in partial integro-differential equations and allow both the modeling of proliferative/destructive events and the modeling of mutations occurring in the onset of tumor, [13]. Further modeling approaches for the immune system-cancer competition include cellular automata, agent-based models, see the recent expository paper [14].

Most of the mathematical models of the IS summarize the response of the immune system in a single population of cells, named effector cells, which perform the task of destroying cancer cells. This simplifying assumption allows to reduce the complexity of the dynamics of immune system but it neglects the recognition process that occurs among the different cells that constitutes the response of the IS to the tumor antigen.

**Figure 1:**
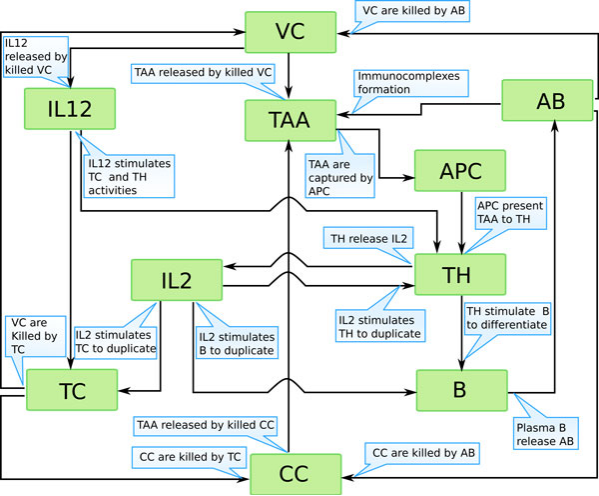
**Conceptual model of the in vivo experiment**. On top vaccine cells (VC) are administered through intravenous injections, and then recognized by Cytotoxic T cells (TC) and Antibodies (AB) that kill them. Killed VC release both Interleukin-12 (IL12) and Tumor associated antigens (TAA). TAA are captured by antigen presenting cells (APC) and then presented to T helper cells (TH). IL12 stimulates both TH and TC actions. TH release interleukin-2 (IL2) which boosts TH, TC, and B actions and stimulates B cells to differentiate into plasma B cells (B). B release AB, and both AB and stimulated TC kill cancer cells (CC), which further release TAA.

The ODE-based model proposed in this paper has been derived from a biological conceptual model that is a good representation of the biological scenario (see Figure 1). The model takes into account both the humoral and cellular response of the immune system and the recognition process that involves the following entities: vaccine cells (VC), cancer cells (CC), tumor associated antigens (TAA), Plasma B cells (B), thymus cytotoxic lymphocytes (TC), thymus helper (TH) lymphocytes, antibodies (AB), interleukins 2 and 12 (IL2 and IL12), and antigen presenting cells (APC). A simplified version of the mathematical model proposed in the present paper has been analytically investigated in [15,16]. The simplified model does not include the role of the associated antigens, plasma B cells, interleukins 2 and 12 and the antigen presenting cells. Therefore the mathematical model of the present paper is a robust extension, from the biological viewpoint, of the model analyzed in [16]. In the present paper we restrict our attention to the comparison of numerical solutions of the model with in vivo experiments and sensitivity analysis of the model parameters. The model described in this paper can be also developed in order to take into account many biological phenomena, like chemotaxis, spatial cell dynamics or cluster formation. If spatial cells dynamics needs to be included, one can use the mathematical framework of the kinetic theory for active particles, see [17] and the reference therein. According to the latter framework, cells are grouped in functional subsystems which express a specific strategy (called activity) and the time evolution of the subsystem is represented by a distribution function over the cells microscopic state (position, velocity and activity). In this framework the mathematical modeling of the chemotaxis phenomenon and the formation of tumor at tissue scale can be included as shown in [18] and [19,20].

The present paper is organized as follows: Section “The Triplex vaccine in vivo experiments” briefly deals with the phenomenological analysis of the biological system. Section “The ODE-based model” is devoted to the description of the ODE-based model. Section “Sensitivity analysis” introduces the sensitivity analysis technique. Section “Results and discussion” compares, by means of numerical simulations, the mathematical model with the computational model SimTriplex. Finally Section “Conclusions” concludes the paper with a critical analysis and research perspective of the model. For interested readers Additional File 1 presents a simplified version of the model by coupling the differential model with an algebraic model.

## Materials and methods

### The Triplex vaccine in vivo experiments

This section briefly deals with the in vivo experiment carried on BALB-neuT neu virgin female mice groups which over-express the activated rat HER-2/neu oncogene. The description does not pretend to be exhaustive from the biological point of view but highlights the essentials of the experiments in order to motivate our study.

The Triplex vaccine has been obtained from a mammary carcinoma of a FVBneuN #202 (H-2*^q^*) mouse, transgenic for the rat protooncogene c-neu, and combines different stimuli:

*• *The p185neu oncoantigen;

*• *The H-2*^q ^*MHC molecules (allogeneic for H-2*^d ^*BALBneuT mice);

*• *The interleukin-12 (vaccine cells are engineered with the genes coding for murine IL-12).

The experiment starts at the sixth week of age, where BALB-neuT mice start the vaccination protocol. Mice are divided in different groups, one for control untreated group, and one for each protocol tested. All vaccine protocols that have been tested are built upon the same 4-week cycle which consists in twice-weekly intra peritoneum vaccinations (Tuesday and Friday) for the first 2 weeks followed by 2 weeks of rest.

The Prophylactic, lifelong Chronic vaccination protocol of cancer-prone HER-2/neu transgenic mice with cells expressing HER-2, allogeneic MHC antigens and IL-12 demonstrated able to completely prevent the onset of mammary carcinoma. The Early vaccination protocol (which counts only three 4-week cycles at the beginning of the experiment) produces a significant delay in the onset of tumors, but all mice eventually succumbe to mammary carcinoma. Other tested protocols demonstrated much less effective, with little or no gain in efficacy when compared to untreated control mice.

It is worth stressing that maximal prevention against mammary carcinoma required all the three vaccine components (HER-2/neu, allogeneic MHC antigens, and IL-12) and was due to the induction of both cellular and humoral immune responses. Although cellular and humoral immune responses are taken into account in the vaccine administration, the relative importance of antibody subclasses for successful cancer prevention indicates that humoral immune responses is more important than cellular responses driven by cytotoxic T cells [4].

**Figure 2:**
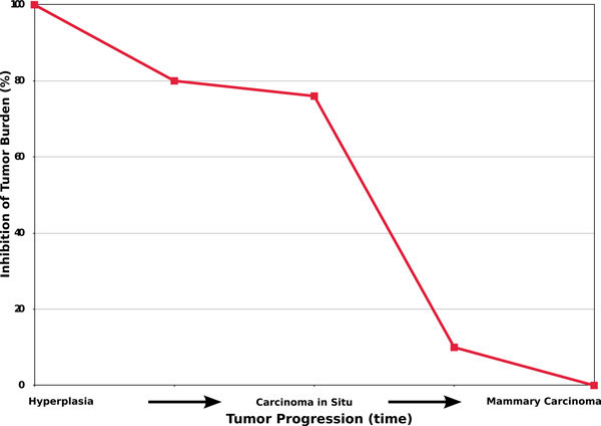
**Triplex vaccine efficacy measured in in vivo experiments with respect to the advancement of the tumor**. The abscissa represents the main temporal stages of tumor progression: from atypical hyperplasia up to mammary carcinoma. The ordinate shows the rate of inhibition of tumor burden entitled with the use of the vaccine. The red line represents the achievable efficacy of the Triplex vaccine in preventing the tumor burden whether the first protocol administration is delayed at successive stages of tumor progression.

Recent investigations [21] show that the Triplex vaccine progressively looses its efficacy with the advancement of tumor progression, both in terms of tumor incidence and multiplicity (see Figure 2). In particular, tumor development is remarkably delayed in mice receiving the early protocol with respect to untreated mice, whereas protocols started later have produced only a negligible delay. Furthermore in vivo tests show that the Triplex vaccine is ineffective against larger tumor targets. Thus, the triplex vaccine demonstrates very effective at preventing mammary carcinoma onset in tumor-free mice but is ineffective against established local tumors.

It should be therefore clear that any vaccination protocol should be started early enough to avoid carcinoma in situ formation. On the other hand it should be advisable to minimize the number of administrations in order to both maintain complete efficacy and reduce the risk of any undesirable effect. In order to help biologists in finding better vaccination protocols, a (multi) agent model named SimTriplex has been developed in [5]. The model has been inspired by the work of Celada and Seiden [22] and uses an approach that models ab initio the interaction and diffusion kinetics of each relevant biological entity. SimTriplex has been tuned with the in vivo experiments and demonstrated able to coherently reproduce the behaviors of the entities involved in the in vivo immunoprevention experiment. In addition the use of SimTriplex as a predictive tool yielded to encouraging results [23].

### The ODE-based model

The competition between immune system cells and cancer cells reminds the well known Predator-Prey (PP) model described by Lotka-Volterra equations. There is a population of prey, represented by the cancer cells, with an infinite set of food resources (nutrients coming from the host blood) and, differently from the classical PP, multiple populations of predators (the effectors cells) cooperate through cell-cell and cell-molecule interactions to neutralize the prey. Differently from the classical predator-prey models, predator survival does not depends on the number of prey, since predator populations exist normally and, in absence of the prey, their number oscillate around given equilibrium levels.

If cancer cells are able to escape immunosurveillance, the cancer takes over, tends to compete with the healthy cells for nutrients, and could be able to kill the host. However the immune system response can be helped in recognizing harmful cells by an external agent represented in our case by a vaccine. The induced immune response is the result of a complex network of interactions between IS cells which mainly depends from cells receptors. IS cells which present, through their receptors, specific tumor antigens can trigger a complex process whose final result is the eradication of the tumor. Specific interactions, which involve cell receptors, cannot be described with an ODE population model, see [5], however if we assume that the vaccine cells activate IS cells at given ratios we can model the subsequent immune response of activated cells.

The network of organs, cells and molecules involved in the immune system is very large. In the model of the present paper we include only the entities recognized as fundamental for the biological process. We also assume that the IS-cancer competition occurs only in one hybrid organ which includes all the physical involved compartments (peritoneum, mammary gland, lymph nodes and so on).

Both the humoral and cellular responses of the immune system are taken into account with their main entities, plasma B cells (B), cytotoxic and helper T lymphocytes (TC and TH), antibodies (AB), antigen presenting cells (APC) and interleukins 2 and 12 (IL2 and IL12), while the pathogens are represented by the cancer cells (CC), the vaccine cells (VC) and the P-185 tumor associated antigens (TAA). The interactions among the various entities of the immune system network, the external stimulus of the vaccine and the cancer cells are modelled by a system of ten nonlinear ordinary differential equations whose variables are summarized in table 1; In Figure 1 we show the conceptual model for the biological problem.

**Table 1:** Model variables

Variable	Description	Short name
*x*_1_	Number of injected vaccine cells	VC
*x*_2_	Number of P-185 tumor associated antigens	TAA
*x*_3_	Number of activated B cells	B
*x*_4_	Number of activated T helper cells	TH
*x*_5_	Number of interleukin 12 molecules	IL12
*x*_6_	Number of interleukin 2 molecules	IL2
*x*_7_	Number of released antibodies	AB
*x*_8_	Number of cancer cells	CC
*x*_9_	Number of activated cytotoxic cells	TC
*x*_0_	Number of activated antigen presenting cells	APC

#### Model parameters

The model contains 44 parameters which have a specific biological meaning. These parameters, assumed as constants, modify the rate of variations of the populations due to natural death, interactions with other population and release of new quantities. Accordingly: parameters referring to natural death of entities are denoted by *μ_i_*, where *i *identifies the population under consideration; parameters referring to the interaction between populations *i *and *j *are identified by *α_i j_*; finally parameters referring to releasing processes are identified by *γ_i j_*, where *i *refers to the released entity and *j *to the releasing entity.

#### Vaccine cells

The vaccine cells dynamics is described by equation (1). Vaccine cells (*VC*) are injected into the host through intraperitoneal vaccination with a predefined dosage. The inoculation of the vaccine cells is modeled by a function *k_in_*(*t, q*) which adds *q *vaccine cells to the cells in the host at time *t *whether a that time an injection was scheduled. Since vaccine cells come from the external, this term represents the only source element in the equation. Vaccine cells die for multiple causes, such as natural death (term -*μ*_1_*VC*), are inhibited by cytotoxic T cells that recognize vaccine cells thanks to their allogenic-MHC class II molecules (term -*α*_19_*TCVC*), or by specific antibodies that are able to directly kill vaccine cells by complement mechanism (term -*α*_17_*ABVC*).

(1) $$\frac{dVC}{dt}=k_{in}\left(t,\;q\right)-\mu_{1}VC-\left(\alpha_{19}TC+\alpha_{17}AB\right)VC$$

#### P-185 tumor associated antigens

Equation(2) models tumor associated antigens dynamics. Tumor associated antigens (*TAA*) can be released by dead or killed vaccine or cancer cells. Accordingly we suppose that the number of released antigens is proportional to both the number of vaccine cells and the number of cancer cells (*VC *and *TAA *respectively) that are inhibited (terms *γ*_21_(*α*_19_*TC*+ *α*_17_*AB*+*μ*_1_)*VC *and *γ*_28_(*α*_88_+*α*_89_*TC*)*VC*). These two terms represent the source elements of the equation. Antigens are subjected to natural degradation (term *μ*_2_*TAA*) and phagocytosis by antigen presenting cells (-*α*_20_*APCTAA*), such as dendritic cells, macrophages and B cells. Moreover antibodies can bind to free antigens producing immune complexes (term -*α*_27_*ABTAA*).

(2) $$\begin{array}{c} \frac{dTAA}{dt}=\gamma_{21}\left(\alpha_{19}TC+\alpha_{17}AB+\mu_{1}\right)VC+\gamma_{28}\left(\alpha_{88}+\alpha_{89}TC+\alpha_{87}AB\right)CC+\\ \;\;\;\;\;\;\;\;\;\;-\;\left(\mu_{2}+\alpha_{20}APC+\alpha_{27}AB\right)TAA \end{array}$$

#### T helper cells

The key role of T helper cells is to stimulate both the humoral and cellular branches of the immune response by direct receptor binding or the releasing of specific cytokines that boost the immune response, such as interleukin 2. T helper cells are activated by specialized APC such as dendritic cells, macrophages or presenting B cells which present major histocompatibility class II/peptide complexes.

Since we are dealing with a monoclonal model, presentation is not directly modeled, so the percentage of activated T helper cells is estimated from the number of antigen presenting cells (*APC*) existing in the system (term *γ*_40_*APC*). Interleukins 12 and 2 (*IL*12 and *IL*2) contribute to stimulate T helper cells priming and duplication $\left(\text{terms}\;\alpha_{46}\;\left(\frac{IL2}{IL2+s_{1}}\right)TH\;\text{and}\;\alpha_{45}\left(\frac{IL12}{IL12+s_{2}}\right)TH\right)$. It is worth noting that, since interleukin 2 is released by T helper cells, these cells are able to self-stimulate their activities. The death factor is modeled by -*μ*_4_*TH *term.

(3) $$\frac{dTH}{dt}=\gamma_{40}APC+\alpha_{46}\left(\frac{IL2}{IL2+s_{1}}\right)TH+\alpha_{45}\left(\frac{IL12}{IL12+s_{2}}\right)TH-\mu_{4}TH$$

#### Plasma B cells

Plasma B lymphocytes may absolve to multiple functions in building the immune response chain against pathogens. In a first stage they can act as specialized antigen presenting cells, by recognizing pathogens through their specialized "Y-shaped" receptors, and can then present peptidic sequences to T helper cells. As a consequence of a successful interaction with T helper cells, they can be stimulated to differentiate into plasma B cells, which release antibodies with the same receptors shape, or B memory cells, which readily act against new appearance of previously encountered pathogens. Since there is no in vivo experimental evidence of B memory cells, as also suggested by the need of a chronic vaccination to achieve complete protection against tumor onset, we decided to do not include for now memory B cells into the model [4].

In equation(4) we only consider the behavior of the B cells population (*B*) that has been activated by T helper cells (*TH*) positive feedback (term *γ*_34_*TH*) and is therefore able to release specific antibodies against cancer cells. We include the B as APC function in equation (10). Interleukin 2 (*IL*2) released by T helper cells plays an adjuvant role in stimulating B cells duplication $\left(\text{term}\;\;\alpha_{36}\left(\frac{IL2}{IL2+s_{3}}\right)B\right)$. Death is modeled by -*μ*_3_B term.

(4) $$\frac{dB}{dt}=\gamma_{34}TH+\alpha_{36}\left(\frac{IL2}{IL2+s_{3}}\right)B-\mu_{3}B$$

#### Interleukin 12

Interleukin 12 (*IL*12) is mainly introduced through vaccine administrations, so it depends on the vaccine dosage. In previous in vivo experiments [24] interleukin 12 was introduced separately, but after transduction of IL2 genes inside vaccine cells [4], it is released by killed vaccine cells, so it is proportional to the number of killed vaccine cells (term *γ*_51_(*α*_19_*TC *+ *α*_17_*AB *+ *μ*_1_)*VC*). IL2 is subjected to normal degradation (-*μ*_6_*IL*12) and it is partially absorbed for mitotic and stimulation signals by cytotoxic and helper T cells priming (terms -*α*_59_*TCIL*12 and -*α*_54_*THIL*12).

(5) $$\frac{dIL12}{dt}=\gamma_{51}\left(\alpha_{19}TC+\alpha_{17}AB+\mu_{1}\right)VC-\left(\alpha_{54}TH+\alpha_{59}TC+\mu_{5}\right)IL12$$

#### Interleukin 2

Interleukin 2 is mainly released by T helper cells (term *γ*_64_*TH*). As previously stated, interleukin 2 stimulates T helper priming, and primed T helper cells produce further interleukin 2. It is subjected to normal degradation (-*μ*_6_*IL*2) and it is partially absorbed for mitotic and stimulation signals in cytotoxic T cells priming (term -*α*_69_*TCIL*2) and B cells duplication (term -*α*_63_*BIL*2).

(6) $$\frac{dIL2}{dt}=\gamma_{64}TH-\left(\alpha_{63}B+\alpha_{69}TC\right)IL2-\mu_{6}IL2$$

#### Antibodies

Antibodies represent the main result of the humoral immune response. Antibodies (*AB*) are released by plasma B (*B*) cells (term *γ*_73_*B*) and are subjected to normal degradation (modeled by -*μ*_7_*AB *term). More-over they disappear in absolving their functions: binding to specific targets, i.e. antigens (term *α*_72_*TAAAB*), cancer and vaccine cells (terms *α*_78_*CCAB *and *α*_71_*VCAB*, respectively).

(7) $$\frac{dAB}{dt}=\gamma_{73}B-\left[\alpha_{78}CC+\alpha_{71}VC+\alpha_{72}TAA\right]AB-\mu_{7}AB$$

#### Cancer cells

Cancer cells growth (*CC*) is modeled through the term $\left[\left(1-\frac{CC}{c_{max}}\right)\;\;k-\alpha_{88}\right]CC$. The term -*α*_88_*CC *is used to take into account CC killing by other immune system cells that are considered of minor importance for the process and are consequently not explicitly modeled, such as Natural Killer cells which can kill cancer cells that under-express the major histocompatibility class I complex. The term *p *models the continuous production of newborn cancer cells. Due to transgenic nature of HER-2/neu mice new cancer cells are in fact continuously introduced into the host. The other terms describe cancer cells death mainly due to antibodies (term -*α*_87_*ABCC*) and cytotoxic T cells (term -*α*_89_*TCCC*) actions. As matter of a fact activated cytotoxic T cells can kill cancer cells by direct cytotoxicity and specific immunoglobulins can kill cancer cells by complement and other mechanisms.

(8) $$\frac{dCC}{dt}=\left[\left(1-\frac{CC}{c_{max}}\right)k-\alpha_{88}\right]CC-\left(\alpha_{89}TC+\alpha_{87}AB\right)CC+p$$

#### Cytotoxic T cells

Cytotoxic T cells priming (*TC*) depends mainly on vaccine cells (*VC*). Vaccine cells are engineered with allogeneic major histocompatibility class I complex in order to easier presentation (term *γ*_91_*VC*). Duplication is instead indirectly stimulated by T helper cells through the release of interleukin 2 $\left(\text{term}\;\;\alpha_{96}\left(\frac{IL2}{IL2+s_{96}}\right)TC\right))$. Natural death is modeled with the term -*μ*_9_*TC*.

(9) $$\frac{dTC}{dt}=\gamma_{91}VC+\alpha_{96}\left(\frac{IL2}{IL2+s_{96}}\right)TC-\mu_{9}TC$$

#### Antigen presenting cells

With the term antigen presenting cells we indicate a class of different types of cells, such as dendritic cells, macrophages, but also B cells, whose focal mission is to recognize, capture, and process antigens in order to present small antigenic sequences named peptides in conjunction with MHC class molecules to both cytotoxic and helper T cells.

Antigen Presenting cells (*APC*) are then depending on the quantity of the antigens that have been released(term *γ*_02_*TAA*), and can die (term -*μ*_0_*APC*).

(10) $$\frac{dAPC}{dt}=\gamma_{02}TAA-\mu_{0}APC$$

We thus designed the following set of ten non linear ODEs that is able to model the considered system of cell populations and interactions:

$$\left\{\begin{array}{c} \frac{dVC}{dt}=k_{in}\left(t,\;q\right)-\mu_{1}VC-\left(\alpha_{19}TC+\alpha_{17}AB\right)\;VC\\ \frac{dTAA}{dt}=\gamma_{21}\left(\alpha_{19}TC+\alpha_{17}AB+\mu_{1}\right)VC+\gamma_{28}\left(\alpha_{88}+\alpha_{89}TC+\alpha_{87}AB\right)CC+\\ \;\;\;\;\;\;\;\;\;\;-\left(\mu_{2}+\alpha_{20}APC+\alpha_{27}AB\right)TAA\\ \frac{dB}{dt}=\gamma_{34}TH+\alpha_{36}\left(\frac{IL2}{IL2+s_{3}}\right)B-\mu_{3}B\\ \frac{dTH}{dt}=\gamma_{40}APC+\alpha_{46}\left(\frac{IL2}{IL2+s_{1}}\right)TH+\alpha_{45}\left(\frac{IL2}{IL2+s_{2}}\right)TH-\mu_{4}TH\\ \frac{dIL12}{dt}=\gamma_{51}\left(\alpha_{19}TC+\alpha_{17}AB+\mu_{1}\right)VC-\left(\alpha_{54}TH+\alpha_{59}TC+\mu_{5}\right)IL12\\ \frac{dIL2}{dt}=\gamma_{64}TH-\left(\alpha_{63}B+\alpha_{69}TC\right)IL2-\mu_{6}IL2\\ \frac{dAB}{dt}=\gamma_{73}B-\left[\alpha_{78}CC+\alpha_{71}VC+\alpha_{72}TAA\right]AB-\mu_{7}AB\\ \frac{dCC}{dt}=\left[\left(1-\frac{cc}{c_{max}}\right)k-\alpha_{88}\right]CC-\left(\alpha_{89}TC+\alpha_{87}AB\right)\;CC+p\\ \frac{dTC}{dt}=\gamma_{91}VC+\alpha_{96}\left(\frac{IL2}{IL2+s_{96}}\right)TC-\mu_{9}TC\\ \frac{dAPC}{dt}=\gamma_{02}TAA-\mu_{0}APC \end{array}\right)$$

Since we consider populations that are activated by vaccine administrations, we set the following initial conditions:

$$VC\left(0\right)\;=TAA\left(0\right)\;=B\left(0\right)\;=TH\left(0\right)\;=IL\text{12}\left(0\right)\;=IL\text{2}\left(0\right)\;=AB\left(0\right)\;=CC\left(0\right)\;=TC\left(0\right)\;=APC\left(0\right)\;=\;0$$

The parameters in the model have been derived from literature, from measurements made during the in vivo experiment and from the SimTriplex model. Some parameters which belong to the class of free parameters that any model has, were chosen into reasonable rages in such a way that the model was able to reproduce in vivo mean survivals for the untreated, early, and chronic vaccinations using a trial and error technique with mean-square evaluation, see table 2.

**Table 2:** Model parameters

Param	Description	Value(estimate)	Ref
*μ*_1_	VC (*VC*) death rate	ln(2) *= *9	In vivo
*α*_19_	VC killing rate by TC cells (*TC*) (killing)	0.001	Estimated
*α*_17_	VC killing rate by AB (*AB*) molecules (killing)	0.001	Estimated
*q*	No. of cancer cells to inject at every vaccine administration	50	SimTriplex

*γ*_21_	released TAA (*TAA*) rate by killed VC	3	Estimated
*γ*_28_	released TAA rate by killed CC (*CC*)	3	Estimated
*μ*_2_	TAA natural degradation rate	ln(2) *= *9	In vivo
*α*_20_	Binding rate between TAA and APC cells	0.0005	Estimated
*α*_27_	Binding rate between TAA and AB (IC formation)	0.00001	Estimated

*γ*_34_	plasma B cells (*B*) activation rate by TH cells (*TH*)	0.05	Estimated
*α*_36_	B stimulation rate by IL2 (*IL*2)	0.0035	Estimated
*s*_3_	B duplication stimulation threshold due to IL2	400	Estimated
*μ*_3_	B cells natural death rate (half life)	ln(2) *= *15	[32]

*γ*_40_	TH cells (*TH*) activation rate by APC (*APC*) cells	0.15	Estimated
*α*_46_	TH cells stimulation rate by IL2 (*IL*2) (duplication)	0.009	Estimated
*s*_1_	duplication stimulation threshold due to IL2	1000	Estimated
*α*_45_	TH cells cells stimulation rate by (*IL*12) IL12 (duplication)	0.009	Estimated
*s*_2_	duplication stimulation threshold due to IL12	1000	Estimated
*μ*_4_	TH cells natural death rate (half life)	ln(2) *= *15	Estimated

*γ*_51_	IL12 molecules release rate by VC	10	SimTriplex
*α*_54_	absorbed IL12 rate by TH cells for mitotic signals	0.00009	Estimated
*α*_59_	absorbed IL12 rate by TC cells for mitotic signals	0.001	Estimated
*μ*_5_	IL12 molecules natural degradation rate	ln(2) *= *9	[33]

*γ*_64_	IL2 release rate by TH	5	Estimated
*α*_63_	absorbed IL2 rate by B cells for mitotic signals	0.0001	Estimated
*α*_69_	absorbed IL2 rate by TC cells for mitotic signals	0.0001	Estimated
*μ*_6_	IL2 molecules natural degradation rate	ln(2) *= *3	Estimated

*γ*_73_	Released AB molecules rate by B cells	3	SimTriplex
*α*_78_	AB - CC binding rate	0.0001	Estimated
*α*_71_	AB - VC binding rate	0.001	Estimated
*α*_72_	AB - TAA binding rate (IC formation)	= *α*_72_	-
*μ*_7_	AB natural degradation rate	ln(2) *= *7	[34]

*c_max_*	CC (*CC*) growth saturation threshold	10^7^	Estimated
*k*	CC duplication rate	0.0226	SimTriplex
*p*	No. of newborn CC due to transgenic nature of mice	3	SimTriplex
*α*_88_	CC death rate due to other IS entities	0.0000001176	Estimated
*α*_89_	CC killing rate by TC cells	0.00004	Estimated
*α*_87_	CC killing rate by AB	0.00004	Estimated

*γ*_91_	TC cells activation rate by VC	0.2	Estimated
*α*_96_	TC cells duplication rate due to IL2	0.05	Estimated
*s*_96_	duplication stimulation threshold thanks to IL2	400	Estimated
*μ*_9_	TC cells natural death rate	ln(2) *= *21	[35]

*γ*_02_	APC (*APC*) activation rate due to TAA (*TAA*)	0.07	Estimated
*μ*_0_	APC natural death rate	ln(2)/15	[36]

During the in vivo experiment, biological dynamics is observed in time slices that are not smaller than eight hours. For this reason, we set the simulation time step equal to (Δ(*t*) = 8 *hrs*). This biological motivation also determined the SimTriplex time-step. The choice of the physical time-step allows to compare the results of the two models. Both models are supposed to simulate the dynamics of entities inside a volume of 1*μl*, which corresponds to a small portion of mammary gland of mice.

### Sensitivity analysis

In order to understand which parameter may be considered fundamental in this process, it is significant to investigate the sensitivity of the model to the alteration of the parameters. Choosing a parameter in a suitable range while retaining fixed the others, represents the classical way to do sensitivity analysis. This methodology clearly owns limitations i.e., results are strongly bounded to the values of fixed parameters, and different sets of values for the fixed parameters may entitle completely different results.

Partial rank correlated coefficients (PRCC) [25] is a statistical approach used to bypass the above mentioned limitations. It works by calculating the partial correlation on rank-transformed data between input (model parameters) and output (entities behaviors). Such a technique does not depend on the values of fixed parameters and permits to vary all the parameters at the same time, allowing to study the influence of input parameters on the model outcomes. Nevertheless the methodology can be in principle easily applied and used with any kind of continuous or discrete model.

The methodology we used to perform sensitivity analysis (LHS-PRCC) is briefly described as follows. The interested reader can found more information about the methodology in [26]. Parameters space is initially sampled using a Monte-Carlo technique. In this case we use a technique named Latin-Hypercube-Sampling (LHS) [27]. The technique divides the random parameter distributions into *N *(where *N *represents the chosen sample size) equal probability intervals that are then sampled. The choice for *N *should be at least *k *+ 1, where k is the number of parameters varied, but usually much larger to ensure accuracy. In our trials we set *N *= 1000.

After sampling an LHS matrix *X *of sampled parameters is built. Each row represents an unique set of variables for the model sampled without replacement.

The model is then solved for each row of X, and the model output values are stored into an output matrix *Y*. Each matrix is then rank-transformed (*X_R _*and *Y_R_*). *X *and *Y *can be used to calculate the Pearson correlation coefficient. *X_R _*and *Y_R _*can be used to calculate the Spearman or rank correlation coefficient (RCC) and the partial rank correlation coefficient (PRCC).

PRCC between an input parameter *x_j _*∈ *X_R_, j *≤ *k *and output *y *∈ *Y_R _*is then computed by considering the residuals $x_{j}-{\hat{x}}_{j}$ and y-ŷ where ${\hat{x}}_{j}$ and  ŷ are given by the following regression models:

$${\hat{x}}_{j}=c_{0}+\overset{k}{\underset{p=1,p\neq j}{\sum }}c_{p}x_{p}\;\;\;\;\text{and}\;\;\;\;ŷ=b_{0}+\overset{k}{\underset{p=1,p\neq j}{\sum }}b_{p}x_{p}$$

## Results and discussion

**Figure 3:**
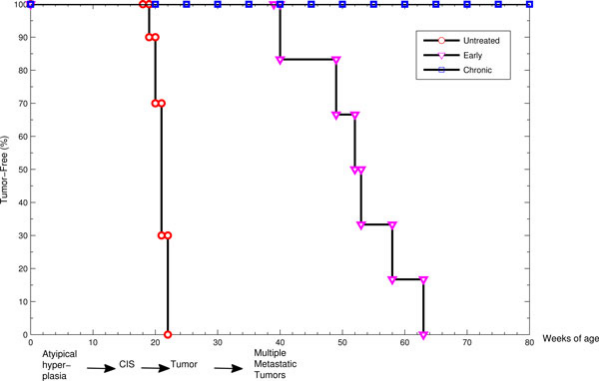
**Kaplan-Meier survival curves**. Kaplan-Meier survival curves given by the in vivo experiment for the Untreated (red circles), Early (purple triangles) and Chronic (blue squares) vaccination protocols.

The outcome of the in vivo experiment has been mainly represented by the mice tumor-free survivals, and the Kaplan Meier survival curves [4] for each mice group treated with a given vaccination protocol have been built accordingly (see Figure 3).

One of the first problems in modeling the process was to determine how to translate the biological concept of death in mathematical/computational terms. When developing the SimTriplex model, it has been decided to stop the simulation and, therefore, to consider a mouse as dead if the total number of cancer cells reached 10^5 ^cells. Over such a threshold the formation of carcinoma in situ can be considered an inevitable circumstance. Since in vivo experiments demonstrated that the vaccine progressively loses its efficacy when such an event occurs [21], this threshold represents a point of no return that halves between survival and death.

Carcinoma in situ formation entitles a lot of different processes such as formation of physical barriers around the tumor mass and vascularization processes that are not described at this stage, even because this goes beyond the scope of the model. This means that both the ODE-based model and the SimTriplex models cannot be considered accurate in describing the in vivo experiment if the cancer cells threshold is overcome. Therefore the numerical simulations presented here refer to interactions where the number of cells do not go beyond this threshold.

As previously stated, the success of failure of a treatment has been determined mainly by the survival rates of the mice involved in the experiment. Even if some measurements were made during the in vivo experiment, it was not possible to keep track of the time evolution of the involved entities. Such measurements are not possible in vivo experiments, or can be achieved just partially in vitro for multiple reasons, i.e. it is not possible to do the measure too frequently due to wet-lab requirements, it is not possible to take the measure at present time with current technology, or simply because the measure entitles the need to kill the host.

One of SimTriplex main features is represented by the possibility of simulating different individuals. Tuning of free parameters has been executed in order to reproduce the same population survival curves for the vaccination protocols tested in vivo [5]. Moreover, during its tuning phase, SimTriplex entities behaviors have been accurately checked by biologists in order to verify that they were qualitatively in line with both biologists assumptions and last immunological knowledge. The use of SimTriplex as a predictive tool, in conjunction with various optimization techniques [28-30], to find better vaccination protocols showed indeed that it represented a good approximation of the in vivo experiment [23], and therefore can be used to substitute missing in vivo data.

Bearing all the above in mind, we initially checked that the mathematical model mice survivals for all the tested vaccine protocols were in tune with mean survivals showed in the in vivo experiment, obtaining a good agreement between the two experiments. For the missing in vivo data, mainly represented by entities time-behaviors, we compared ODE behaviors obtained numerically with the ones obtained by SimTriplex, highlighting similarities and differences.

We would note here that, in order to compare the results, we looked in SimTriplex for "mean virtual mouse", i.e. a mouse whose death occurs near the middle of the Kaplan Meier curves for the tested protocols.

The ODE model demonstrated able to reproduce the available in vivo experimental data, in particular the in silico mice survivals for all vaccine protocols tested were in good agreement with mean survivals showed in in vivo experiment.

Since the biological behavior of the involved entities may change in a consistent manner even from mouse to mouse, we mainly focused in qualitatively analyzing cancer cells behaviors and the response times of the principal outcomes of immune response, i.e. antibodies and cytotoxic T cells behaviors for the Chronic, Early and Untreated protocols.

**Figure 4:**
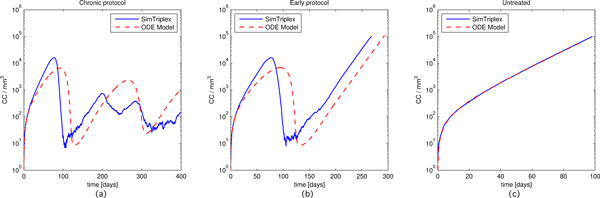
**Number of cancer cells (***CC***) behaviors for the Chronic, Early and Untreated protocols**. Blue solid lines identify SimTriplex simulations, red dashed lines the ODE model numerical results. Plots are presented on a log-scale to improve comparison.

In Figure 4 we compare the number of cancer cells (*CC*) behavior for the three protocols. As the Figure 4 shows, there is a slight delay between SimTriplex and the ODE model curves for both Chronic and Early protocols, whereas the Untreated protocol exhibits negligible difference, since both models use the same parameters for the growth law. Such a delay remains in line with in vivo experiment expectations. Indeed the behaviors are qualitatively in agreement, suggesting that the cancer cells dynamics is well described by the ODE model. The Chronic protocol (see Figure 4, left panel) plot suggests that after an initial growth phase, cancer cells are kept under control from the immune system thanks to the repeated administration of the vaccine.

The above behavior does not happen in the Early case (see Figure 4, center panel), where the vaccination protocol is only able to delay the development of the cancer, and the threshold on the number of cancer cells that entitles high risks of carcinoma in situ is reached at around at 44 weeks of age in SimTriplex, and at 47 weeks in the ODE model. In in vivo experiments the middle of the Kaplan Meyer survival curve for the early protocol is reached approximately at 52 weeks of age [4], with carcinoma in situ formation between 5 to 9 weeks earlier.

**Figure 5:**
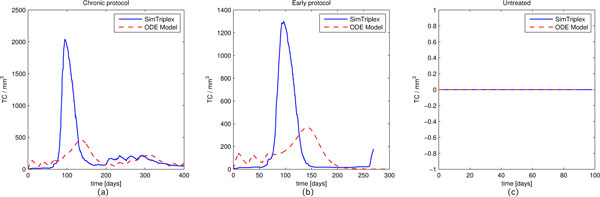
**Number of cytotoxic T cells (***TC***) behaviors for the Chronic, Early and Untreated protocols**. Blue solid lines identify SimTriplex simulations, red dashed lines the ODE model numerical results.

The number of cytotoxic T cells (*TC*) behavior is shown in Figure 5. The untreated plot (see Figure 5, right panel) is flat for both the models since in absence of vaccination there is no cytotoxic T activation. Even in this case we observe that the ODE model plots for the Chronic and Early vaccine protocols are a little bit delayed with respect to the SimTriplex plots, even if they remain in the expected range of the in vivo experiment. This could partially justify the delays observed in the cancer cells plots for the ODE plots (see Figure 4, left and center panels).

Moreover the cytotoxic T cells peaks observed in SimTriplex for both the Chronic and Early protocols (see Figure 5, left and center panels) are a lot higher than those showed by the ODE model. In in vivo experiments it was observed that antibodies covered a major role in eradicating the tumor, whereas cytotoxic activity was estimated to be of secondary importance [23]. So from this point of view the ODE model may indeed be more accurate in describing this aspect of the immune response.

**Figure 6:**
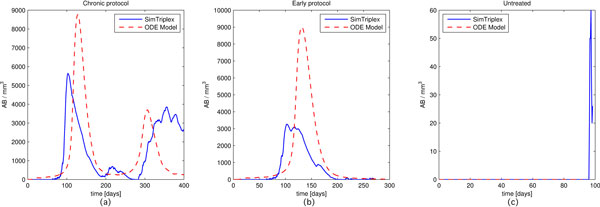
**Number of antibodies (***AB***) behaviors for the Chronic, Early and Untreated protocols**. Blue solid lines identify SimTriplex simulations, red dashed lines the ODE model numerical results.

Finally we analyze the number of antibodies (*AB*) behavior in Figure 6. The untreated plots (see Figure 6, right panel) are practically flat for both the simulations and show negligible difference. Only at the end of the experiment SimTriplex plots show the appearing of antibodies. This may be due to the presence in SimTriplex of Natural killer cells that are able to kill cancer cells which under-express the MHC molecules, giving rise to a week response at late stages. For the Chronic and Early protocols (Figure 6, left and center), the antibodies time-behavior is very similar, but with higher AB peaks in the ODE model than those observed in SimTriplex simulations. This can be seen as a consequence of the weaker cytotoxic immune response observed in the ODE model which requires, in accord with in vivo observations, a stronger humoral immune response in order to deplete the tumor.

We used PRCC to analize the effects of the most important input parameters which influence more the behavior of Cancer Cells. We plotted for these entities the PRCCs over the entire time course of the experiment to how the parameters sensitivity varies as the process behavior advances. The analysis has been executed by supposing that the administration of the vaccine follows the Chronic protocol. In this way it is possible to study which mechanisms mainly drive the immune response against cancer cells and which parameters should be tweaked in vivo in order to obtain a strong immune response with the minimal effort. To this end we kept constant the parameter related to the quantity of injected vaccine cells (*q*) and the parameters related to the tumor growth (*k, p, c_max_*).

**Figure 7:**
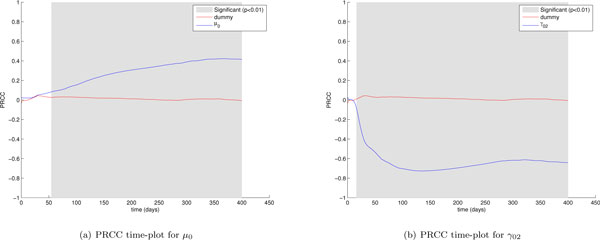
***μ*_0 _and *γ*_02 _PRCC time plots**. Partial Rank Correlated Coefficients are computed on the number of cancer cells (*CC*), and are plotted over time (blue lines). PRCC plot of Dummy parameter (red lines) is presented for comparison. The plot portions where the correlation becomes significant (*p <*0.01) are shown in gray.

From the LHS-PRCC analysis we found that 15 parameters that correlated significantly with the number of cancer cells. For some parameters a negative or positive correlation was somewhat expected, for example it is trivial to observe that the dead rate and the activation rate of APC (*μ*_0 _and *γ*_02_) positively and negatively correlate with cancer cells behavior, respectively (see Figure 7). The time correlation of some parameters indeed brings out some interesting findings we show as follows.

**Figure 8:**
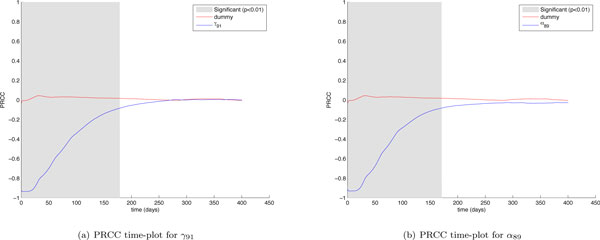
***γ*_91 _and *α*_89 _PRCC time plots**. Partial Rank Correlated Coefficients are computed on the number of cancer cells (*CC*), and are plotted over time (blue lines). PRCC plot of Dummy parameter (red lines) is presented for comparison. The plot portions where the correlation becomes significant (*p <*0.01) are shown in gray.

**Figure 9:**
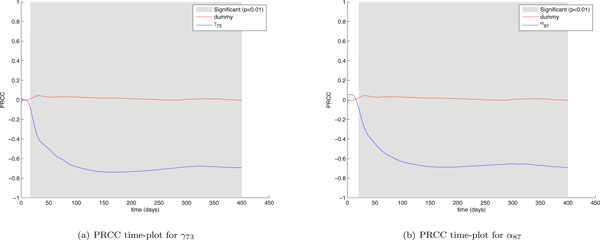
***γ*_73 _and *α*_87 _PRCC time plots**. Partial Rank Correlated Coefficients are computed on the number of cancer cells (*CC*), and are plotted over time (blue lines). PRCC plot of Dummy parameter (red lines) is presented for comparison. The plot portions where the correlation becomes significant (*p <*0.01) are shown in gray.

The first interesting finding is related to the mechanisms driving the immune response against the tumor. The cytotoxic immune response against the tumor is influenced by parameters such as *γ*_91 _which represents the vaccine cells killing rate and α_89 _which represents the cancer cells killing rate by cytotoxic T cells. By taking a look at *γ*_91 _and α_89 _(Figure 8) PRCC time plots it is possible to observe that the two parameters show a strong negative correlation just at the beginning of the experiment. The correlation becomes weaker and weaker as time goes on, becoming totally not significative starting from (around) day 160. The humoral immune response instead is driven by parameters such as *γ*_73 _which represents the antibodies release rate and *α*_8_7 which represents the cancer cells killing rate by antibodies. These parameters show a strong negative correlation which grows fast and lasts up to the end of the experiment (see Figure 9). This means that after an initial stage needed to start the immune response, variations of cytotoxic T cells related parameters do not influence cancer cells behavior, thus suggesting how cytotoxic T cells are not fundamental for the complete eradication of the Tumor, which is instead strongly correlated with humoral immune response related parameters for all the time length of the experiment. This fact confirms the observation made by Palladini et. Al. [23], where in vivo observations showed that the immune response against the tumor was mainly driven by antibodies.

**Figure 10:**
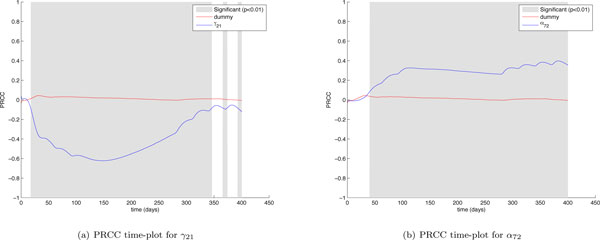
***γ*_21 _and *α*_72 _PRCC time plots**. Partial Rank Correlated Coefficients are computed on the number of cancer cells (*CC*), and are plotted over time (blue lines). PRCC plot of Dummy parameter (red lines) is presented for comparison. The plot portions where the correlation becomes significant (*p <*0.01) are shown in gray.

Another interesting finding can be derived by taking a look at *γ*_21 _and *α*_72 _PRCC time plots (see Figure 10). The *γ*_21 _parameter represents the antigens release rate by vaccine cells whereas the *α*_72 _parameter represents the rate of interaction between antibodies and antigens which brings to the formation of immune-complexes. The *γ*_21 _plot clearly shows a negative correlation with the number of cancer cells. However it is interesting to observe that this correlation becomes weaker at the end of the experiment when the vaccine protocol is used to maintain under control the number of cancer cells, thus suggesting that the role of antigens becomes less important at the end of the experiment. In addition the *α*_72 _PRCC plot shows a positive correlation with the number of cancer cells, suggesting that a higher interaction rate between antibodies and antigens negatively influences the effects of the treatment. This becomes more evident at the end of the experiment (just around day 300) when the last 4 vaccination cycles are administered. Every vaccination cycle seems to reinforce the correlation between the input and the output parameters, affecting negatively the immune response. This can be explained by the fact an higher interaction rate between antibodies and antigens would entitle that more antibodies are recruited in binding antigens, and then fewer antibodies (which as discussed earlier play a major role in the immune system response against the tumor) are employed in killing cancer cells. When the number of cancer cells is kept under control, antigens may enter in competition with cancer cells and may negatively influencing the immune response. From the sensitivity analysis we can conclude that the antigenic administration should be then stronger during the first phases of the immune response, and then reduced once the humoral immune response is well established in order to reduce the risk that too many antibodies are involved in binding antigens instead cancer cells. This model speculation is in line with the biological effect of high antigen stimulation that usually suppresses the immune response [31].

## Conclusions

The mathematical model proposed in this paper is based on nonlinear ordinary differential equations. The model simulates the competition between the immune system and the mammary carcinoma under the action of an external force field (the vaccine). Three different protocols of the vaccine have been taken into account: Untreated, Early, and Chronic. The biological role of vaccine cells, cancer cells, tumor associated antigens, plasma B cells, thymus cytotoxic lymphocytes, thymus helper lymphocytes, antibodies, interleukins 2 and 12, and antigen presenting cells has been taken into account.

Numerical simulations of the model have been performed for different vaccination protocols and results were compared with a previously developed multi-agent model, called SimTriplex. For the tested vaccination protocols, the ODE-based model is able to qualitatively reproduce the time evolution not only for the number of cancer cells, but also for antibodies and cytotoxic T cells, main outcomes of humoral and cell mediated immune responses. From a quantitative point of view the mathematical model showed, respectively, a weaker and a stronger immune response of cytotoxic T cells and antibodies with respect to the SimTripex model, showing indeed better agreement with the in vivo observations and speculations.

The sensitivity analysis gave two major results. First it confirmed the major role of humoral immune response also observed in in vivo experiments [23], then showed that during later stages of the experiment antigens loose their role of activating the immune response and in some cases may negatively influence the immune response. It is then possible to conclude that a reduction of the intensity of vaccine administrations in later stages, when the immune response is already set, is advisable. This has been also highlighted in [8], where an ABM model developed to illustrate the effects of the same vaccine in cancer immunotherapy, suggested to apply the golden standard vaccination procedure (initial boost followed by sparse recalls) also to cancer vaccines.

These results are certainly useful to research activity in immunology addressed to improve the efficacy of the treatment and to modulate the activation of the immune system in order to prevent side effects such as autoimmune diseases. Of course, different choices of initial conditions and of the parameters may modify the competition dynamics.

We plan to investigate the optimal protocol using mathematical tecniques which are currently under investigation. Results will be pubblished in due course.

## Competing interests

The authors declare that they have no competing interests.

## Authors' contributions

CB: Provided mathematical expertise and analytical solution, wrote the paper.

FC: Performed sensitivity analysis, wrote the paper.

FP: Designed the model, analyzed data, supervised the project, wrote the paper.

MP: Designed the model, analyzed data, performed numerical simulations and sensitivity analysis, wrote the paper.

## Supplementary Material

Additional File 1 **Additional File 1 -- On the coupling of differential and algebraic models**. Click here for file
