# Supplementary material for: Mathematical modeling of the immune system recognition to mammary carcinoma antigen
Source: BMC Bioinformatics. 2012 Dec 7;13(Suppl 17):S21. doi: 10.1186/1471-2105-13-S17-S21 (PMC3521211; doi:10.1186/1471-2105-13-S17-S21)
Supplement: Additional File 1 — On the coupling of differential and algebraic models. [file 1471-2105-13-S17-S21-S1.pdf]

Additional File 1: On the coupling of differential and algebraic models

The ODE-based model 1 can be analytically solved under the following considerations. The variables which modify the response of the immune systems are the vaccine cells, the cancer cells, and the APC response. Therefore we can assume that the others variables are time functions depending on VC, CC, and APC.

$$\begin{cases} \frac{dVC}{dt} = k_{in}(t,q) - \mu_1 VC - (\alpha_{19} TC + \alpha_{17} AB) VC \\ \frac{dTAA}{dt} = \gamma_{21}(\alpha_{19} TC + \alpha_{17} AB + \mu_1) VC + \gamma_{28}(\alpha_{88} + \alpha_{89} TC + \alpha_{87} AB) CC + \\ \qquad -(\mu_2 + \alpha_{20} APC + \alpha_{27} AB) TAA \\ \frac{dB}{dt} = \gamma_{34} TH + \alpha_{36} \left(\frac{IL2}{IL2+s_3}\right) B - \mu_3 B \\ \frac{dTH}{dt} = \gamma_{40} APC + \alpha_{46} \left(\frac{IL2}{IL2+s_1}\right) TH + \alpha_{45} \left(\frac{IL12}{IL12+s_2}\right) TH - \mu_4 TH \\ \frac{dIL12}{dt} = \gamma_{51}(\alpha_{19} TC + \alpha_{17} AB + \mu_1) VC - (\alpha_{54} TH + \alpha_{59} TC + \mu_5) IL12 \\ \frac{dIL2}{dt} = \gamma_{64} TH - (\alpha_{63} B + \alpha_{69} TC) IL2 - \mu_6 IL2 \\ \frac{dAB}{dt} = \gamma_{73} B - [\alpha_{78} CC + \alpha_{71} VC + \alpha_{72} TAA] AB - \mu_7 AB \\ \frac{dCC}{dt} = \left[\left(1 - \frac{CC}{c_{max}}\right) k - \alpha_{88}\right] CC - (\alpha_{89} TC + \alpha_{87} AB)\ CC + p \\ \frac{dTC}{dt} = \gamma_{91} VC + \alpha_{96} \left(\frac{IL2}{IL2+s_{96}}\right) TC - \mu_9 TC \\ \frac{dAPC}{dt} = \gamma_{02} TAA - \mu_0 APC \end{cases} \tag{1}$$

Accordingly we propose the following assumptions:

$$\alpha_{19} TC + \alpha_{17} AB = \alpha_1 \tag{2}$$

$$\alpha_{89} TC + \alpha_{87} AB = \overline{k}\tilde{\alpha_2} = \alpha_2 \tag{3}$$

$$\alpha_{20} APC + \alpha_{27} AB = \alpha_3 \tag{4}$$

$$\alpha_{63} B + \alpha_{69} TC = \alpha_4 \tag{5}$$

$$\alpha_{54} TH + \alpha_{59} TC = \alpha_5 \tag{6}$$

$$\alpha_{78} CC + \alpha_{71} VC + \alpha_{72} TAA = \alpha_6 \tag{7}$$

$$IL12 = \alpha_7 \tag{8}$$

$$IL2 = \alpha_8 \tag{9}$$

where α_i, for $i \in \{1,2,3,4,5,6,7,8\}$, and $\tilde{\alpha_2}$ are real constants depending on the parameters of the model and the rate of the injection is assumed constant, $k_{in}(t) = \overline{k}$. We note that $\alpha_2 = \alpha_{89}TC + \alpha_{87}AB$ is defined as a constant proportional to $\overline{k}$ (rate of injection) as in equation (**??**) and it is used to model the IS (cytotoxic and humoral) response against cancer cells, which is stimulated by the vaccine. Moreover, if we assume $\alpha_{19} = \alpha_{89}$, $\alpha_{17} = \alpha_{87}$, and $p = 0$ then the algebraic system (12-19) can be solved as function of VC, CC, and TC. The system (12-19) thus reads

$$AB = \frac{\alpha_1 - \alpha_{19}TC}{\alpha_{17}} \tag{10}$$

$$TH = \frac{\alpha_6 - \alpha_{59}TC}{\alpha_{54}} \tag{11}$$

$$B = \frac{\alpha_4 - \alpha_{69}TC}{\alpha_{63}} \tag{12}$$

$$TAA = \frac{\alpha_6 - \alpha_{78}CC - \alpha_{71}VC}{\alpha_{72}} \tag{13}$$

$$APC = \frac{\alpha_3\alpha_{17} - \alpha_{27}\alpha_1 + \alpha_{19}\alpha_{27}TC}{\alpha_{17}\alpha_{20}} \tag{14}$$

where the functions VC, CC, and TC are the solutions of the following nonlinear Cauchy problem:

$$\begin{cases} \frac{dVC}{dt} = \overline{k} - \alpha VC \\ \frac{dCC}{dt} = (\epsilon - \lambda)CC - \frac{k}{c_{max}}CC^2 \\ \frac{dTC}{dt} = \beta VC + \gamma TC - \delta TC \\ VC(0) = TC(0) = 0, CC(0) = CC^0 \neq 0 \end{cases} \tag{15}$$

where $\alpha = \mu_1 + \alpha_1$, $\beta = \gamma_{91}$, $\gamma = \alpha_{96}\frac{\alpha_8}{\alpha_8 + s_{96}}$, $\delta = \mu_9$, $\epsilon = k$, and $\lambda = \alpha_{88} + \alpha_2$.

The Cauchy Problem (15) has the following analytical solution:

$$VC(t) = \frac{\overline{k}}{\alpha}\left[1 - e^{-\alpha t}\right], \tag{16}$$

$$TC(t) = \frac{\overline{k}\beta}{\alpha}\left[\frac{e^{(\gamma-\delta)t} - 1}{\gamma - \delta} + \frac{e^{-\alpha t} - e^{(\gamma-\delta)t}}{\gamma - \delta + \alpha}\right], \tag{17}$$

and

$$CC(t) = \frac{1}{e^{-(\epsilon-\lambda)t}\left(\frac{1}{CC^0} - \frac{C}{(\epsilon-\lambda)}\right) + \frac{C}{\epsilon-\lambda}}, \tag{18}$$

where $C = \frac{k}{c_{max}}$. Therefore replacing Equations (16), (17), and (18) into Equations (10)-(14) we have the analytical solution of the Differential-Algebraic model.
